# Taxonomy of approaches to developing interventions to improve health: a systematic methods overview

**DOI:** 10.1186/s40814-019-0425-6

**Published:** 2019-03-12

**Authors:** Alicia O’Cathain, Liz Croot, Katie Sworn, Edward Duncan, Nikki Rousseau, Katrina Turner, Lucy Yardley, Pat Hoddinott

**Affiliations:** 1 Medical Care Research Unit, Health Services Research, School of Health and Related Research (ScHARR), University of Sheffield, Regent Court, 30 Regent Street, Sheffield, S1 4DA UK; 2 NMAHP Research Unit, University of Stirling, Stirling, FK9 4NF UK; 3 Population Health Sciences, Canynge Hall, 39 Whatley Road, University of Bristol, Bristol, BS8 2PS UK

**Keywords:** Intervention development, Review, Methodology, Guidance, Health

## Abstract

**Background:**

Interventions need to be developed prior to the feasibility and piloting phase of a study. There are a variety of published approaches to developing interventions, programmes or innovations to improve health. Identifying different types of approach, and synthesising the range of actions taken within this endeavour, can inform future intervention development.

**Methods:**

This study is a systematic methods overview of approaches to intervention development. Approaches were considered for inclusion if they described how to develop or adapt an intervention in a book, website or journal article published after 2007, or were cited in a primary research study reporting the development of a specific intervention published in 2015 or 2016. Approaches were read, a taxonomy of approaches was developed and the range of actions taken across different approaches were synthesised.

**Results:**

Eight categories of approach to intervention development were identified. (1) Partnership, where people who will use the intervention participate equally with the research team in decision-making about the intervention throughout the development process. (2) Target population-centred, where the intervention is based on the views and actions of the people who will use it. (3) Evidence and theory-based, where the intervention is based on published research evidence and existing theories. (4) Implementation-based, where the intervention is developed with attention to ensuring it will be used in the real world. (5) Efficiency-based, where components of an intervention are tested using experimental designs to select components which will optimise efficiency. (6) Stepped or phased, where interventions are developed with an emphasis on following a systematic set of processes. (7) Intervention-specific, where an approach is constructed for a specific type of intervention. (8) Combination, where existing approaches to intervention development are formally combined. The actions from approaches in all eight categories were synthesised to identify 18 actions to consider when developing interventions.

**Conclusions:**

This overview of approaches to intervention development can help researchers to understand the variety of existing approaches, and to understand the range of possible actions involved in intervention development, prior to assessing feasibility or piloting the intervention. Findings from this overview will contribute to future guidance on intervention development.

**Trial registration:**

PROSPERO CRD42017080553.

**Electronic supplementary material:**

The online version of this article (10.1186/s40814-019-0425-6) contains supplementary material, which is available to authorized users.

## Background

Policy makers, health professionals, patient groups, the public, designers and researchers develop interventions, programmes or innovations to improve health. It is important that the intervention development process maximises the chances that an intervention will be effective and sustainable. Unless it does, there is a risk of research waste [1], where expensive evaluations are undertaken of flawed interventions that turn out not to be feasible, acceptable or effective in subsequent feasibility studies or fully powered evaluations [2].

In recent years, researchers have published journal articles, websites and books on how to develop interventions. This international endeavour, proposing ways of developing interventions that others can follow, could be described as the production of guides, guidance, methodology or frameworks. In this article, the umbrella term ‘approaches’ is used. These approaches are distinct from publications describing the development of a specific intervention. Approaches that show how to develop interventions are useful for those new to intervention development. They offer an opportunity for research communities to refine and improve those approaches for future use.

There are a variety of approaches to intervention development and it is timely to bring these together and synthesise them to understand the range of actions available. Previous reviews of intervention development have focused on identifying approaches used in the specific context of behaviour change in implementation science [3], optimisation in terms of making final modifications to interventions prior to formal evaluation [4], the use of theory in intervention development for a single condition [5] and ways of adapting interventions for ethnic minority communities [6].

Complex interventions are widely used to improve health. These interventions have multiple interacting components, target multiple groups or levels of an organisation and attempt to affect multiple outcomes [7]. The United Kingdom Medical Research Council (MRC) is widely cited for its guidance on developing and evaluating complex interventions [7], describing the four phases of developing, feasibility/piloting, evaluation and implementation. One part of the guidance has been extended recently to offer more detail on process evaluation [8]. Guidance on the feasibility/piloting phase is currently being extended, following recent publication of a systematic review of existing guidance for this phase [9]. Some researchers have considered enhancements to the development phase of the MRC guidance for the specific field of nursing studies [10]. The MRC has funded a study to produce guidance on intervention development: ‘IdentifyiNg and assessing different approaches to DEveloping compleX interventions (the INDEX study). As part of the INDEX study [11], a systematic review of approaches to intervention development was undertaken to identify the range of approaches available, and to synthesise the actions within these approaches, in order to help researchers to develop complex interventions and to inform future guidance on intervention development.

## Methods

### Systematic methods overview

Systematic methods overviews are reviews of the methods literature [12–14]. Guidance has been published to help researchers to undertake systematic methods overviews [14]. This guidance was followed to undertake a systematic methods overview of different approaches to developing complex interventions. Exhaustive searching and inclusion of all relevant literature associated with systematic reviews of primary research is not necessary because learning and arguments about methodology and methods are repeated frequently in the literature. Instead, there is an emphasis on broad searching to identify the range of relevant literature, and on data saturation of learning and arguments [12, 13]. The protocol is available, registered at PROSPERO CRD42017080553.

### The aim of the overview

The aim of this overview was to identify a broad range of approaches to intervention development. The emphasis was on recently produced or recently used approaches, because of the rapid development of this field, with newer approaches building explicitly on older approaches. The objectives were to construct a taxonomy of approaches to help future developers think about the approach they might take, and to synthesise the actions within each approach to identify the full range of actions developers can consider.

### Definitions used in this overview

#### Intervention

A health intervention is an effort, activity or combination of programme elements designed to improve health status. This overview focuses on complex interventions that include a number of components which may act both independently and inter-dependently. This includes policy innovations such as introducing a new health service or public health policy nationally (e.g. smoking ban in public places). It does not include the development of medicines and any invasive interventions (e.g. pills, procedures, devices). Complex interventions to improve health or health care outcomes can be delivered in many settings including health care facilities, schools, local communities or national populations. They can be delivered by a range of individuals including health care, social care and public health practitioners, as well as professionals working outside of the health care sector, such as teachers, charity workers and peers.

#### Intervention development

Craig et al. [15] proposed the development phase to be the period when the ‘intervention must be developed to the point where it can reasonably be expected to have a worthwhile effect’. (p. 9). The start and end points of the development phase are not always clear. There may be overlap between the development phase and the subsequent phase of feasibility and piloting, because some exploration of feasibility is often part of the intervention development process [16]. A helpful indicator of the end of the development phase is the production of a document or manual describing the intervention and how it should be delivered [16]. There may also be overlap between the intensive development phase and a longer period of preparation prior to intervention development, when a team undertakes a series of studies over a number of years before the point of formally developing an intervention. This may involve assessment of the evidence base, including reviewing the effectiveness of existing interventions, and/or qualitative research with stakeholders. Alternatively, these studies may be undertaken as part of the intensive intervention development phase. This overview focuses on the intensive development phase, recognising that the start and end of this phase may be hard to define.

#### Refinement, optimisation, modification and adaptation

**Fig. 1:**
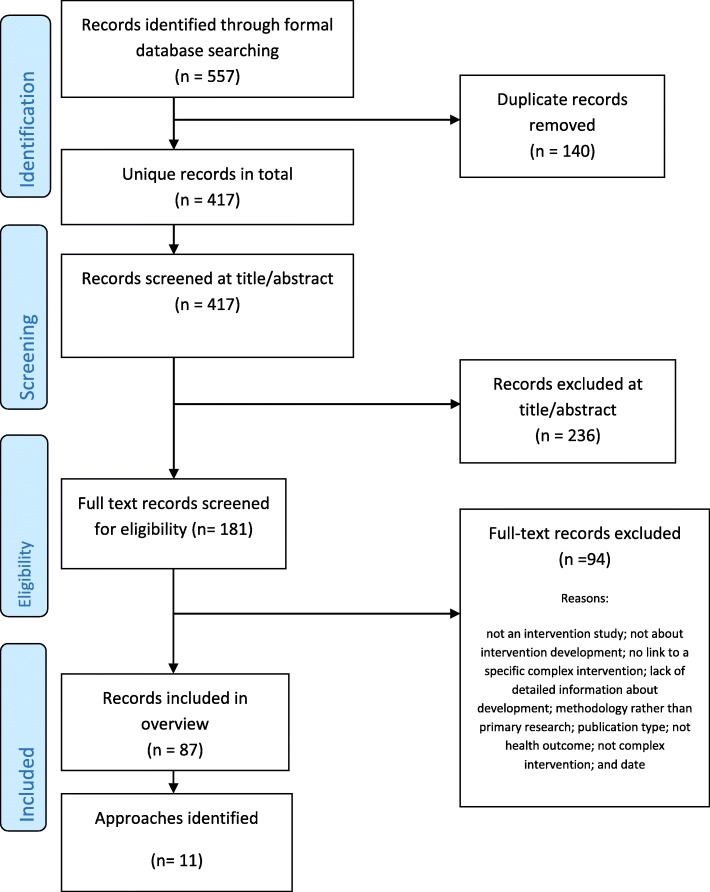
PRISMA 2009 flow diagram: search for primary studies only

During the development process, the initial version of the intervention may be repeatedly refined by making improvements based on early assessment of feasibility and acceptability. This process continues throughout the formal feasibility/pilot testing and evaluation phases (see Fig. 1). Indeed, some researchers see intervention development as a long-term ongoing endeavour which lasts throughout the full evaluation and implementation phases [17]. Early refinement, during the development phase, is included in this overview but later refinement, during or after the formal pilot phase of an evaluation, is excluded.

Intervention optimisation is the process of improving the efficiency of an intervention. Different components are assessed to identify those affecting intermediate outcomes, so that only effective components are included in the intervention to be fully evaluated. A recent review of optimisation of complex health interventions prior to a full trial has been published [4]. Approaches to optimisation are therefore not a focus of this overview unless their authors frame them specifically as approaches to intervention development.

Sometimes researchers take interventions that have been shown to be effective at the evaluation phase, and perhaps implemented in the real world, and adapt them for a new sub-population, health condition or context (Fig. 1); for example, an existing effective intervention might be adapted for ethnic minority communities [6]. Such adaptation may involve a formal development phase, so approaches to adaptation are included in this overview if they are framed by their authors as intervention development.

#### An ‘approach’ to intervention development

‘Approach’ refers to the whole process of intervention development documented in a book, website or journal article where authors explicitly offer a guide to undertaking intervention development. Approaches may provide different amounts of detail about how to develop an intervention. All are included in this overview regardless of the amount of detail offered.

#### Development versus design

Sometimes researchers use the terms ‘development’ and ‘design’ interchangeably. In this overview, the term ‘development’ is used for the whole process of intervention development and the term ‘design’ is reserved for a point in the development process where developers make decisions about the intervention content, format and delivery.

### Search

The focus of systematic methods overviews can include the literature describing or critiquing methods or methodology, and the methods sections of primary research papers [14]. The focus of this overview is recent literature documenting how to develop an intervention. Approaches were considered for inclusion if they describe how to develop an intervention in a book, website or journal articles published after 2007, or are cited in a primary research study reporting the development of a specific intervention published in 2015 or 2016.

In systematic methods overviews, the search strategy should be transparent and broad rather than exhaustive [13, 14]. The process started with a primary search of the databases Medline, CINAHL, PsycINFO, ASSIA and ERIC from January 2015 to December 2016 using the single search term ‘intervention development’. These health, social science and education databases were selected because they include research on complex interventions with health outcomes. Title and abstract screening, followed by full text search, identified journal articles reporting primary research of the development of specific interventions. The methods sections of these articles were read by KS and AOC in order to identify any intervention development approaches that were used and referenced. The most up-to-date version of journal articles, books or websites referenced in these articles were obtained for data extraction. This search also identified articles describing approaches, and systematic reviews of approaches, to intervention development. Because the search term ‘intervention development’ was simple and potentially limited the breadth of approaches identified, a check was undertaken with a second search using a broader set of search terms to see if this yielded a broader set of approaches. A set of diverse terms associated with intervention development were searched in the same databases in the same time period: complex behavioural intervention, develop, design, phase I, exploratory, refine and translate. This identified 808 records. AOC and KS conducted a title and abstract screen on a sample of records from this search: the first 100 records and 1 in every 8 records. Allowing for overlap, this identified 189 records. The full texts of 26 met the inclusion criteria and did not identify further approaches to intervention development. Both search strategies are detailed in Additional file 1.

Searching should go beyond standard bibliographic databases because methodologies/methods are described in books as well as in journal articles [14]. The formal search described above was supplemented by a search in Google Scholar using the terms ‘intervention development’, ‘complex intervention development’, ‘intervention optimisation’, ‘complex intervention pre-clinical’, ‘intervention adaptation’ and ‘intervention modification’. Different terms to those used in the searches of databases were used deliberately to facilitate broad searching. Finally, the authors of this overview drew on their existing reference libraries because use of personal knowledge is also important in reviews of complex evidence [18].

### Inclusion and exclusion criteria

Approaches were considered for inclusion if they presented a guide to developing an intervention that had been produced or updated since 2007 or used in primary research published 2015–2016. A purposeful approach to selection of literature is advocated in systematic methods overviews, with use of maximum variation sampling. The inclusion of literature stops when new issues no longer emerge. As approaches were identified, members of the team (AOC, LC, KS) inductively developed a taxonomy of approaches. Data saturation was considered and further approaches not included if the team considered that saturation was reached. For example, not all approaches to developing digital interventions were included because they repeated the actions already identified within that category of approach. Attention was also paid to diversity of context within each category and across all categories in the emerging taxonomy. This process was led by AOC with team discussions with LC and KS.

### Data extraction

For each approach, AOC read the article, website or book and extracted the rationale stated by authors for the approach, the context for which the approach was constructed, the key actions undertaken, the methods used to deliver each action and the strengths and limitations of the approach. These strengths and limitations were identified by the authors of the approach, the authors of other approaches included in the overview or the research team (indicated by ‘INDEX’).

### Quality appraisal

Quality appraisal is a challenge in these overviews [14]. There was no formal assessment of the quality of the approaches to intervention development because assessment criteria do not exist.

### Analysis

The constant comparative method has been recommended for synthesising within systematic methods overviews [14]. Most overviews are aggregative in terms of bringing together different concepts, rather than interpretative in terms of developing new concepts [14]. A constant comparative aggregative approach was undertaken within three concurrent steps:

(i) AOC extracted data on rationale, context, key actions, methods and strengths and limitations to summarise each approach within a table.
(ii) AOC developed a taxonomy of approaches. AOC grouped approaches together based on the stated rationales for each approach because these convey the intentions of the authors. These rationales were extracted from statements made by the authors when introducing their approach. The categories of approach, and the individual approaches included within them, were discussed and refined by AOC, LC and KS until an agreed taxonomy was produced.
(iii) AOC listed the actions from each approach, grouped similar actions and brought these together to identify a comprehensive set of actions from all the categories of approach, including the methods that could be used at each action. AOC, LC and KS discussed these actions until agreement was reached.

## Results

### Approaches identified and included

As previously stated, the intention was to undertake a broad rather than an exhaustive search, where more informal searches are as important as formal searches. PRISMA flow charts are devised to display exhaustive searches within standard systematic reviews. For this systematic methods overview, a PRISMA flow chart is displayed for the search using the term ‘intervention development’ of primary studies reporting intervention development in 2015–2016 (Fig. 1).

### Taxonomy of approaches

Eight categories of approach to intervention development were identified, distinguished by the rationales stated by the authors of these approaches (Table 1). The review team identified the following labels for these eight categories based on the language used by authors of approaches.

1. Partnership intervention development. Three approaches were included, addressing a range of contexts (quality improvement, social innovation and radical innovation). Partnership approaches included co-production with equal participation in decision-making of the research team and the people whom the intervention aimed to help, and user-driven development. Due to similarities with approaches already included, a partnership approach for implementation science was not included [19]. Primary research studies reported using community-based participatory research but articles or books describing how to use this approach for intervention development could not be located so it was not included here.
2. Target population-centred intervention development. Three approaches were included, addressing a wide range of contexts (health care delivery, technology, behaviour change and self-management).
3. Evidence and theory-based intervention development. Six approaches were included, addressing a range of contexts (complex interventions in health and health care, public health, social policy, behaviour change and quality improvement). Some of these approaches also proposed a rationale of being systematic (see category 6) but were included in this category because they emphasised the role of evidence and theory within their rationale. There were a large number of these. Some approaches identified in the primary research study search were not included here due to data saturation [20, 21].
4. Implementation-based intervention development. One approach was included, in the context of health behaviour interventions.
5. Efficiency-based intervention development. Three approaches were included, although they were not independent of each other. Two of the approaches defined different ways of optimising components for parts in the first approach in this category.
6. Stepped or phased-based intervention development. Three approaches were included, addressing a range of contexts (public health, social policy and clinical practice).
7. Intervention-specific development. Five approaches were included in three intervention groups—digital behaviour change interventions, patient decision aids and group interventions. Other approaches were identified but not included here because of data saturation, because they were outside the time range of post 2007 and had not been used in the primary research studies published 2015–2016 [22], or because they offered recommendations for the future rather than current guidance [23].
8. Combination approach to intervention development. There was one approach included here in the context of behaviour change.

**Table 1:** Taxonomy of approaches to intervention development

Category	INDEX team definition	Defined approach	Source
1. Partnership	The people for whom the intervention aims to help are involved in decision-making about the intervention throughout the development process, having at least equal decision-making powers with members of the research team	Co-production, co-creation, co-design, co-operative design	Voorberg et al. 2015 [38] Bessant and Maher 2009 [39] Spencer et al. 2013 [40]
		User-driven	Kushniruk and Nøhr 2016 [25]
		Experience-based co-design (EBCD) and accelerated EBCD	Robert et al. 2013 [41] Locock et al. 2014 [42]
2. Target population-centred	Interventions are based on the views and actions of the people who will use the intervention	Person-based	Yardley et al. 2015 [17]
		User-centred	Erwin and Krishnan 2016 [44] Erwin and Krishnan 2016 [45] Erwin 2015 [43–45]
		Human-centred design	Norman 2013 [33]
3. Theory and evidence-based	Interventions are based on combining published research evidence and formal theories (e.g. psychological or organisational theories) or theories specific to the intervention	MRC Framework for developing and evaluating complex interventions	MRC Guidance [7, 15]
		Behaviour change wheel (BCW)	Michie et al. 2014 [26]
		Intervention mapping (IM)	Bartholomew Eldredge et al. 2016 [27]
		Matrix Assisting Practitioner’s Intervention Planning Tool (MAP-IT)	Hansen et al. 2017 [32]
		Normalisation process theory (NPT)^a^	Murray et al. 2010 [46]
		Theoretical domains framework (TDF)	French et al. 2012 [47]
4. Implementation-based	Interventions are developed with attention to ensuring the intervention will be used in the real world if effective	Reach, Effectiveness, Adoption, Implementation, Maintenance (RE-AIM)	RE-AIM.org [48]
5. Efficiency based	Components of an intervention are tested using experimental designs to determine active components and make interventions more efficient	Multiphase optimization strategy (MOST)	Collins et al. [49]
		Multi-level and fractional factorial experiments	Chakraborty 2009 [50] Dziak et al. 2012 [50, 51]
		Micro-randomisation trials	Klasnja et al. 2015 [52]
6. Stepped or phased based	Interventions are developed through emphasis on a systematic overview of processes involved in intervention development	Six essential Steps for Quality Intervention Development (6SQUID)	Wight et al. 2015 [28]
		Five actions model	Fraser and Galinsky 2010 [29] Fraser et al. 2009 [24, 29]
		Obesity-Related Behavioural Intervention Trials (ORBIT)	Czajkowski et al. 2015 [34]
7. Intervention-specific	An intervention development approach is constructed for a specific type of intervention	Digital (e.g. Integrate, Design, Assess and Share (IDEAS))	Mummah et al. 2016 [30] Horvarth et al. 2016 [30, 53]
		Patient decision support or aids	Elwyn et al. 2011 [31] Coulter et al. 2013 [31, 54]
		Group interventions	Hoddinott et al. 2010 [55]
8. Combination	Existing approaches to intervention development are combined	Participatory Action Research based on theories of Behaviour Change and Persuasive Technology (PAR-BCP)	Janols and Lindgren 2017 [56]

^a^Could be considered under implementation based approaches to intervention development because the theory is about implementation

The stated rationale, context, key actions and strengths and limitations of each included approach are described in Table 2 and these characteristics are considered below.

**Table 2:** Description of different approaches to intervention development

Category	Approach	Rationale	Context specified by authors	Steps, activities or actions specified by authors^a^	Strengths specified by authors of approach, authors of other approaches and the overview team INDEX (source in brackets)	Limitations
1. Partnership	Co-creation, co-production, co-design [38]	Active involvement of end users in various stages of the production process produces more effective and efficient services with higher user satisfaction [38, 40] A key issue is an equal relationship between the end users (and their families and communities) and professionals, with shared decision-making [40]. It can also be seen as ‘user-led innovation’ [39]. Requires a shift in power from professionals to community or end users [40] Co-creation produces sustainable competitive advantage [39] Customises solutions to specific contexts [39] Delivers services appropriate to the needs of patients and advances equality [40]	Quality improvement in health and social care Social innovation in public sector services Radical innovation—as opposed to incremental—in health services	Six steps: 1. Identify and build an initial team including end users and people important to the service, developing inclusive communication processes. Use joint and equal involvement of staff, patients, researchers, people leading improvement, and design professionals 2. Define and share assets—knowledge, experience, skills and abilities, influence and connections. Understand the current problem through non-participant observation, patient interviews, log books, films, local press, use of cameras, workshops, storytelling, etc. 3. Co-create the vision by listening to all voices 4. Co-design the solution using qualitative research, rapid ethnography and prototyping. Use tools to generate creative thinking. Open up a range of potential solutions as described in user and human centred approaches below. 5. Build the solution possibly using small action groups who can use their relevant expertise. Make use of prototyping methods. 6. Measure outcomes together and plan this as an integral part of the process	There are examples of changes made to services based on this approach [25] and reductions in the cost of health care provision [40] Studies what people do rather than what they say they do [39] A detailed guide is available [40]	Attention has not been paid to the outcomes of co-creation [38] Quantitative methods need to be used because qualitative evaluation of co-creation is dominant [38] [38] is a systematic review of the use of co-creation discussing different levels of involvement of end users including co-design (the developers lead the process in partnership with the end users), co-implementation (end users implement a service with formal service providers) and initiation (end users develop and implement innovation). They offer insights into the process rather than a tool-kit (INDEX)
	User-driven [25]	A participatory approach goes beyond user-centred design, with users as active participants in generating design ideas and decision-making. In co-operative design, users and designers work together to come up with a design and further refinements. In user-driven design, the users lead the creative thinking and the designers facilitate the process. End user involvement is critical to the adoption of information systems because it increases functionality and the quality of the system Involvement empowers users	Information systems in health	Proposes three levels of participation in design: user-centred (see next group in this table), cooperative (see co-production earlier) and user-driven. Important activities include: 1. Establish co-operation between users and designers 2. Gain insight into current problems and needs and generating visions for future solutions. This may involve ‘design games’ to free minds and creativity. 3. Continual and iterative input from end users 4. Develop prototypes and undertake usability testing of them in real life environments or a simulation of this to identify interactions with wider users and activities affecting use. 5. Bring the users who were observed using the prototypes into further design meetings for active participation in refinement of prototype	Can be low cost and rapid and thus increase dissemination of new designs [25] Shown to be successful at improving future prototypes and preventing the introduction of systems that fail [25]	How, when and where to engage users remains open to question [25] Ensuring the users involved are representative of the target population is challenging [25] Reaching consensus when there are differing voices is challenging [25] Difficult for clinical staff to give time for design but there are ways of working rapidly to alleviate this [25] The ‘interventions’ are not necessarily intended for evaluation in an RCT but may be used immediately in the real world (INDEX)
	Experienced based co-design (EBCD) and accelerated experience based co-design (AEBCD) [41, 42]	Need in-depth patient experience (narrative) to take action and make improvements to services Patient accounts generate priorities and solutions that service providers may not think of Patient narratives can help patients and staff reflect on how to improve services and establish an emotional connection between staff and patients Patients as equal partners in co-design can generate improvement AEBCD is more feasible than EBCD in the complex cash-strapped real world and offers a rigorous and effective approach to quality improvement	Service improvement specific to a single service in a single setting	Core ‘strands’ are: -Participatory action research -User centred -Reflective practice -Narrative There are six steps in two phases: Phase 1 Discovery 1. Project management established 2. Local staff are interviewed about their experiences. 3. Local patients are interviewed about experiences to produce a ‘trigger film/video’ to prompt discussion amongst patients and staff about improvements needed. In AEBCD, the film is based on a national archive rather than gathering local patient experiences. Patients and carers are invited to view the video and identify priorities. Phase 2 co-design where family, patients and staff are equal partners in small working groups 4. The priorities of staff and patients, and the video, are considered by patients, carers and staff in a workshop meeting to identify priorities for improvement. 5. Small co-design groups established to implement improvements. 6. Small groups re-convene to celebrate and review progress.	Draws on rigorous narrative-based research with a broad sample of patients rather than a narrow group of people [42] Active partnership between patients and staff and focus on tangible results produces results [42] Evaluation shown to be successful at producing improvements in the target service and in wider aspects of the hospital [42] Hospitals commit investment to doing this again so they see it as successful [42] Online training toolkit is available (INDEX)	Discovery phase is time consuming so not practical in real world of health care. Therefore, AEBCD preferable [42] Some patients found the video more negative than their own experiences; there was a heavy workload for local facilitators but they obtained wider benefits such as capacity building [42] Useful for local service improvement rather than developing a generalizable intervention (INDEX)
2. Target population based	Person-based approach [17, 57]	Enhances acceptability and feasibility of an intervention at early stages of development and evaluation Systematic investigation In depth understanding of users leads to interventions that are more relevant, persuasive, accessible and engaging Complements theory-based and evidence-based design Matches fundamental design to needs and goals of users	Digital health-related behaviour change interventions and illness management interventions because people use e-health independently Has also been used outside digital interventions for self-management Behaviour change interventions Early stages of development and evaluation	Uses mixed methods research and iterative qualitative studies to investigate beliefs, needs, attitudes and context of target population Two elements: First, a developmental process using qualitative research with a diverse sample of target population. Goes beyond acceptability, usability and satisfaction to understand the psycho social context of the user so can make intervention relevant to them. Second, identify ‘guiding principles’ to guide intervention development. These elements are used at four stages of the process: 1. At the planning stage undertake synthesis of qualitative studies or qualitative research to prioritise what is important or identify new components of an intervention 2. At the design stage identify the intervention objectives and features of the intervention required to deliver them 3. When the prototype is available, evaluate acceptability and feasibility 4. Implement in real life setting to further modify intervention	Systematic way of gaining in depth understanding of users’ perspectives to make the intervention more relevant and engaging [17, 57] Shown to be successful because interventions have been effective in RCTs [17, 57] Advantage over co-design is that people are basing views on actual use of the intervention [17, 57] Different from user-centred approach used in computer-based research because looks beyond usability and technical issues [17, 57] Reasonable amount of detail given, with examples (INDEX)	Iterative approach may be hard to respond to quickly in practice [17, 57]
	User-centred design [43]	Making delivery more efficient and equitable by putting people at the centre of any problem to develop solutions that better fit their everyday lives, activities and context Must design interventions to fit users’ needs and context to facilitate translation of evidence into the real world May need new approaches to address complexity	Innovation in organisations Improving health care delivery	Early and continuous stakeholder engagement, including having stakeholders as part of research team to undertake contextual inquiry. Three phases: Phase I Defining design requirements: Use of role play and observation to identify issues rather than only qualitative interviews; develop prototypes to get specific views on the intervention Phase II Develop a prototype and refine in iterative interviews: e.g. rank priority of concepts; converse with stakeholders to improve fit Phase III Evaluate stakeholder preferences: e.g. compare with alternatives and get quantitative feedback, card sorting of statements to obtain views	Multi-stakeholder driven [44, 45] Focuses on what users and practitioners actually do, not simply on what they say they do [44, 45] Shifts focus from content of intervention to delivery in context so helps to overcome barriers to implementation in the real world [44, 45] Uses prototypes to get specific rather than generic feedback [44, 45] Focus is on utility, fit and engagement of key users of the intervention [44, 45]	Although there is a book as well as journal articles, more details could be given about how to achieve each action (INDEX)
	Human-centred design [33]	Study people and take their needs and interests into account so that technology and appliances meet the needs of people including that it is enjoyable and useable	Design of machines, appliances, technology for everyday use Not health	Four activities are proposed, working within a multidisciplinary team: 1. Observing—Philosophy of early focus on observing the target users and tasks rather than asking users what they want. Good designers do not start by trying to solve the proposed problem but by trying to understand what the real issues are. 2. Ideation—Consider a wide range of potential solutions and be creative 3. Prototyping—build quick rough prototypes to continue to understand the problem 4. Testing and undertaking rapid testing of ideas/prototypes with the target population in real circumstances and modifying approach after each iteration Throughout, consider wider issues such as the cost of the object or stigma Attached to using it	The focus on the starting point of the process, and not closing down questioning and ideas too early are important actions not articulated well in other approaches (INDEX)	Working within time, budget and other constraints [33]
3. Theory and evidence based	MRC Framework for developing and evaluating interventions [7, 15]	Spending time developing interventions systematically based on evidence and theory produces interventions which have a reasonable chance of having a worthwhile effect	Complex interventions in health care, public health and social policy	Three functions: 1. Identifying the evidence base 2. Identifying/developing theory 3. Modelling process and outcomes Questions are also identified for researchers to ask themselves, such as ‘Have you used this theory systematically to develop the intervention?’ and ‘Can you describe the intervention fully, so that it can be implemented properly for the purposes of your evaluation, and replicated by others?’	Not prescriptive [7] Well cited and used in grant proposals [58] Used by many researchers in primary research (INDEX)	Little detail [28, 47], INDEX Issues were under intense development and debate at time of writing guidance [7] Lacks attention to complexity science [58]
	Behaviour Change Wheel (also action by action approach) [26]	Comprehensive and systematic approach, encouraging designers to consider the full range of options through systematic evaluation of theory and evidence	Behaviour change interventions in health and can be used in other settings	Eights steps in three stages: 1. Understanding the behaviour i. Define the problem in behavioural terms ii. Select the behaviours you are trying to change iii. Specify the target behaviour, i.e. who needs to do what differently and when iv. Identify what will bring about the desired behaviour change using COM-B or Theoretical Domains Framework 2. Identify intervention options that will bring about change i. Identify intervention functions ii. Identify policy categories 3. Identify content and implementation options i. Identify behaviour change techniques from list of 93, e.g. goal setting ii. Identify mode of delivery	As well as aiding intervention design it improves evaluation and theory development by helping to understand why interventions have failed or how they have worked [26] Explicitly draws attention to the different levels at which an intervention may need to work [26] Clear and detailed explanation of each action with multiple examples ([32], INDEX) Well known [32] Popular in that used by many researchers in primary research (INDEX)	Acknowledges that judgements are required where there is no evidence but does not say who should be involved in making these judgements e.g. stakeholder groups (INDEX) Although reference is made to working with stakeholders, the emphasis is on behaviour change (INDEX) Needs more emphasis on the target population being involved in process [56] Requires substantial knowledge of psychological processes [32]
	Intervention mapping [27]	A systematic and thorough approach using theory and evidence will produce an effective intervention	Health promotion Public health Complex problems	Addresses planning, implementation and evaluation. 6 steps: 1. Undertake a needs assessment to develop a logic model of the problem 2. Produce a logic model of the change process that leads to outcomes 3. Design the scope, sequence, methods and practical applications of the program 4. Produce the program including the materials 5. Plan implementation and maintenance of the program 6. Develop an evaluation plan Using with a community-based participatory approach may help external validity	Extremely rigorous and elaborate approach to intervention development ([28], INDEX) Used by many researchers [32] and cites a long list of published interventions developed with this approach (INDEX—see p34–38 of book) Addresses environmental as well as personal factors affecting the problem [32]	Highly technical, prescriptive, can require years to implement, and difficult to operationalise [28] Does not cover the full range of intervention options available [26] So comprehensive that it requires time resources that make it unfeasible for use by many developers [32]
	Matrix Assisting Practitioner’s Intervention Planning Tool (MAP-IT) [32]	Making the use of theoretical knowledge and empirical evidence easy can help practitioners to develop effective interventions at low cost	Health promotion Behaviour change complex health interventions	A matrix is determined by a small group of expert researchers focused on a specific behaviour change for a specific age group, e.g. promoting physical activity in older adults. The experts create a matrix of personal and environmental mechanisms that promote positive behaviour, relevant theories and functions of an intervention that could address each mechanism. This matrix can then be used by practitioners to develop a theory-driven and evidence-based intervention	It undertakes one part of intervention development for behaviour change so that developers do not have to understand psychological theory in depth (INDEX) Links scientific research with practical real world applications [32] Offers a feasible and low cost approach for practitioners developing interventions [32] Synthesises concepts in other well-known approaches [32]	One matrix is presented here. Matrices need to be produced for other conditions/risk factors in a variety of age groups [32] It is insufficient because it does not take the context in which the intervention will be used into account [32] It facilitates one part of intervention mapping rather than offering a full approach to intervention development (INDEX)
	Normalisation Process Theory (NPT) [46]	Using theory about normalising interventions in routine practice can help develop and evaluate interventions that will be implemented in the real world if found to be effective	Complex interventions in health and health care	The components of the theory can help to 1. Describe the context in which the proposed intervention will be implemented 2. Define the intervention using literature reviews, observation, interviews and surveys	Focuses on wider system issues and interactions between different groups of staff and patients, addressing both individual and organisational level factors [46] Addresses a neglected aspect of intervention development (INDEX)	Focuses on one aspect of intervention development (INDEX) No detail about how to develop interventions (INDEX)
	Theoretical Domains Framework (TDF) [47]	Using a theoretical framework in a systematic way to develop an intervention will help to make hypothesised mechanisms of change explicit and change clinical practice	Complex interventions Clinical behaviour change Implementation interventions to get evidence into practice Quality improvement	A four-step systematic method based on guiding questions: 1. Who needs to do what, differently? 2. Which barriers and enablers need to be addressed (using a theoretical framework)? 3. Which components could overcome modifiable barriers and enhance enablers? 4. How can behaviour change be measured and understood?	A conceptual aid and not a rigid prescription [47] Uses theory, evidence and mixed methods research [47] Using a broadly based theoretical framework for behaviour change is better than using a single theory [47]	Requires considerable time and resources but spending this time and resource may be a good investment [47] No detail about to how to undertake each action (INDEX)
4. Implementation-based	Reach, Effectiveness, Adoption, Implementation, Maintenance [48]	To encourage intervention planners and other stakeholders to pay more attention to external validity to improve the sustainable adoption and implementation of effective interventions To help plan interventions and improve their chances of working in ‘real-world’ settings. To facilitate translation of research to practice	Health behaviour interventions	The RE-AIM Planning Tool [48] is a series of questions which serve as a checklist for key issues to consider when planning an intervention. The questions are within five groups: 1. Planning to improve reach to the target population 2. Planning for effectiveness 3. Planning to improve adoption by target staff, settings, or institutions 4. Planning to improve implementation 5. Planning to improve maintenance of intervention effects in individuals and settings over time	The approach has been used to evaluate and report a wide range of interventions [48] The emphasis on developing interventions that will be used in the real world if effective is complementary to some existing approaches to intervention development (INDEX)	RE-AIM [48] was originally developed as a framework for consistent reporting of research results and then as a framework for evaluating interventions. As such, there is little detail about how to develop interventions (INDEX)
5. Efficiency-based	Multiphase Optimization Strategy (MOST) [49]	Conceptually rooted in engineering, MOST emphasises efficiency and careful management of resources to move intervention science forward systematically Randomised experimental approaches to optimisation leads to more potent interventions	Multicomponent behavioural interventions in public health	There are three phases: 1. Preparation: information from sources such as behavioural theory, scientific literature and secondary analyses of existing data is used to form the basis of a theoretical model. 2. Optimisation: randomised experiment to test the effectiveness of different components. Fractional factorial experiments (see below) sequential multiple-assignment randomised trials (SMARTs) or micro-randomised trials (see below) may be used here. 3. Evaluation: standard RCT. A continuous cycle of optimisation and evaluation can occur	A number of projects using MOST have been funded by national funding agencies [49]	Focuses on a narrow aspect of intervention development, occurring after the components of the intervention have been assembled or designed (INDEX)
	Multi-level and fractional factorial experiments [50, 51]	Simultaneous screening of candidate components of an intervention to test for active components offers an efficient way of optimising interventions	Multi component interventions with behavioural, delivery or implementation factors and where there is clustering	Conduct a ‘screening experiment’ to determine which components go forward to experimental evaluation. Starts with a number of potential components and removes the least active ones. Uses fractional factorial design to screen out inactive components rather than evaluate the utility of a combination of components over a single component. Focuses on main effects and a few anticipated two-way interactions	Superior to mediational analyses from first RCT followed by second RCT [50]	Lack of statistical power to do this at the development phase (INDEX) Focuses on a narrow aspect of intervention development, occurring after the components of the intervention have been assembled or designed (INDEX)
	Micro-randomised trials [52]	Delivering the right intervention components at the right times and locations can optimise support to change individuals’ health behaviours	‘Just in time adaptive interventions’ (mobile health technologies) Behaviour change	Multiple components are randomised at different decision points for an individual. An individual may be randomised hundreds of times over weeks or months. Intermediate outcomes can be measured rather than primary outcomes		Only suitable for some types of intervention where participants are prompted to do something, where events are common and where measurement of intermediate outcome is low burden [52] Focuses on a narrow aspect of intervention development, occurring after the components of the intervention have been assembled or designed (INDEX)
6. Stepped/phased	Six essential Actions for Quality Intervention Development (6SQuID) [28]	To guide researchers Practical, logical, evidence based approach to maximise effectiveness of interventions To reduce waste of public money by not evaluating useless interventions	Public health but authors say wider relevance	1. Define and understand problem and its causes 2. Identify which causal or contextual factors are modifiable, and which have the greatest scope for change 3. Identify how to bring about change (the mechanisms of action) 4. Identify how to deliver the mechanisms of change 5. Test and refine the intervention on a small scale 6. Collect enough information about effectiveness to proceed to full evaluation	Systematic, logical and evidenced to maximise likely effectiveness [28] Practical guidance where none exists [28] Attention to both early and later stages of the development process (INDEX) Based on experience of development and evaluation of interventions (INDEX)	Offers an overview rather than detail (INDEX) Although authors recommend taking some of the actions with involvement from stakeholders, and using qualitative research at later stages, little attention needed to involvement of those receiving and delivering the intervention (INDEX)
	Five action model in intervention research for designing and developing interventions [24, 29]	A systematic process of developing a manual leads to interventions that change practice A detailed manual allow replication of effective interventions	Social work Social and public health programs Based on developing and testing interventions in child development	The focus is on creating the intervention and then refining it during evaluation There are five steps: 1. Develop both problem theory and program theory: specify the problem, the rationale for the intervention and the theory of change 2. Design intervention materials to articulate strategies for changing malleable mediators. Develop first draft of manual specifying the format of manual (content, order of content and who delivers it). Revisions and adaptations to the manual occur throughout the further actions. 3. Refine and confirm program components in efficacy tests. Submit manual for review by relevant stakeholders including target population and those delivering the intervention. Undertake mixed methods feasibility testing. 4. Test effectiveness in a variety of practice settings 5. Disseminate program findings and materials	Specifies link between the problem theory and the intervention content [29] Specifies process of developing treatment manuals [29]	The five actions cover evaluation as well as development so there is not as much detail about the development stage as in other approaches (INDEX) Although practitioners are considered early in the process, the target population is considered late in the process of development (INDEX)
	Obesity-Related Behavioural Intervention Trials (ORBIT) [34]	A systematic, progressive framework for translating basic behavioural science into treatments that address clinical problems in a way that strengthens the treatments and encourages rigorous evaluation	Clinical Behavioural treatments for preventing and treating chronic diseases	Flexible and progressive process making use of iterative refinement and optimisation. The five steps are: 1. Identification of a significant clinical question 2. Phase 1a Design: Develop a hypothesised pathway from behaviour treatment to a solution for the clinical problem 3. Phase 1b Refine: Optimise content and delivery of an intervention, and tailor to sub-groups 4. Phase IIa Proof of concept: When treatment manual is available, undertake study on small numbers to see if it merits more rigorous and costly testing 5. Phase IIb Pilot testing: Look for benefits achieved over and above a control group or consider the feasibility of a full evaluation	Clinically relevant and uses language from drug development to appeal to medical stakeholders [34] Constructed for use with a broad number of chronic diseases rather than a single category of disease [34] Details milestones needed at the end of one phase prior to moving on to next phase (INDEX)	Takes a similar approach to MRC Guidance by using the phases of drug trials in an iterative phased approach. Only focuses on the first phases of drug trials and although there is more detail about development than the MRC guidance, there is still a lack of detail compared with other approaches (INDEX)
7. Intervention-specific	Digital: IDEAS (Integrate, Design, Assess, and Share) Framework for digital interventions for behaviour change [48]	Guiding intervention development using the best combination of approaches helps to deliver effective digital interventions that can change behaviour Need a *combination* of behavioural theory and user-centred design thinking to develop effective interventions. These must be evaluated and disseminated to maximise benefit	Digital Behaviour change	Covers development and evaluation. Ten phases in four stages 1. Integrate insights from users and theory i. Empathise with target users ii. Specify target behaviour iii. Ground in behavioural theory 2. Design iteratively and rapidly with users iv. Ideate implementation strategies v. Produce prototype vi. Obtain user feedback vii. Create a product 3. Assess rigorously viii. Pilot test to assess potential efficacy and usability ix. Evaluate in RCT 4. Share x. Share intervention and results	Offers action by action guide about combining behaviour theory and design thinking [48] Strikes a balance between offering sufficient detail without being overly prescriptive [48]	Less experienced users may find it difficult to apply [48] There may be disagreements amongst team members that are challenging to manage [48]
	Digital—practical advice for internet-based health interventions [53]	Concrete examples from experience of digital intervention development can complement best practices guidance	Online health interventions Public Health	Based on the views of researchers and practitioners: 1. Hire the right research team, e.g. include computer science experts 2. Know the needs of the target population 2. Plan the process before engaging a web designer 3. Recognise that different stakeholders have different values and language e.g. researchers and web designers 4. Develop a detailed contract 5. Document all decisions 6.Use a content management system 7. Allow extra time for testing and refining	Based on views of researchers with experience and offers complementary knowledge of intervention development to existing published sources [53]	The focus is largely on how to work with commercial web designers in the context of a digital intervention (INDEX)
	Web-based decision support tools for patients [31]	A clear project management and editorial process will help to balance different priorities of variety of stakeholders [31] Need close consultation with target users and iterative development process to develop accessible and useful intervention [31]	Decision aids available in web-based versions	A process map for developing decision aids addressing two areas: First, content specification by combining scientific evidence and patient perspectives. Second creative design to tailor it to specific audiences by considering presentation of information, help for patients to assess how they feel about future events and allow patients to formulate a preference Five groups are established: a project management group of 3–4 people to drive the process; an advisory group of 6–10 stakeholders who advise but do not have editorial rights; a virtual scientific reference group of experts to review evidence synthesis and the evolving tool; a technical production group which will create and host the website; and stakeholder consultations with a series of prototypes including patients undergoing the decision and practitioners who interact with patients Overlapping steps are: 1. Identify patients’ needs using qualitative research 2. Evidence synthesis 3. Consensus on evidence 4. Construct storyboard 5. Undertake sandpit testing with experts 6. Undertake usability testing 7. Undertake field testing with real patients	Use of creative design and consultation as well as scientific evidence [31] Close liaison with target users [31] Iterative method of refinement [31]	Time consuming [31] One action dependent on earlier actions so can be delays [31] Can be disagreements between experts, and between health professionals and patients [31]
	Patient decision aids [54]	Systematic and transparent process of development allows users to check validity and reduce chance of causing harm and increase chance of benefit. Explicit that there is no hard evidence to support this rationale	Decision support	Based on a review of different approaches to developing decision aids, core features common to all are: 1. Scoping and design 2. Development of a prototype 3. Iterative ‘alpha’ testing by patients, clinicians and other stakeholders involved in the development 4. Iterative ‘beta’ testing in real-life contexts with patients and clinicians not involved in the development 5. Production of final version The process is overseen by a multi-stakeholder group	More comprehensive than previous guides [54]	Uncertainty remains about how best to address the individual elements of the guide [54] Lack of detail about how to undertake different actions (INDEX)
	Group interventions [55]	More systematic approach to designing interventions	Health improvement interventions or behaviour change interventions occurring in a group setting in public health and primary care	Interventions are complex adaptive social processes with interactions between the group leader, participants, and the wider community and environment. When designing them consider: 1. What the intervention is and the quantity delivered 2. How someone becomes a group member 3. The social and behavioural theories that inform the intervention 4. How the group influences members’ attitudes, beliefs and behaviours. Existing theories may inform this, e.g. social support theory 5. The intended outcomes 6. Who should be the target population	Fills a gap in the evidence base [55] Can be used in conjunction with another approach when delivery to groups is required (INDEX)	Framework also covers evaluation so there is a lack of detail about development (INDEX) Details issues to think about rather than how to develop the intervention (INDEX)
8. Combination	Participatory Action Research process based on theories on Behaviour Change and Persuasive technology (PAR-BCP) [56]	Aids the integration of theories into a participatory action research design process because behaviour is hard to change	Behaviour change systems for health promotion (possibly in digital health)	Combines theory from two fields (behaviour change and persuasive technology) with a participatory action research methodology. A checklist includes 1. Understand and define the behaviour to target 2. Understand the target group’s experiences and attitudes towards the behaviour and intervention 3. Consider ease of use of intervention 4. Understand what kind of proactive feedback is needed to change behaviour 5. Understand how to visualise progress 6. Explore what about the patient-health professional relationship builds trust 7. Describe how social interactions can promote behaviour change 8. Evaluate prototypes	Brings together two categories of approach to intervention development: partnership and theory-based (INDEX)	No detail on how to undertake actions (INDEX) Although the label ‘participatory action research’ is used, some examples describe a target population centred approach (INDEX)

^a^These actions are summaries and readers are advised that source documents should be read to understand the detail of each approach

### Contexts

The approaches included were produced for a wide range of contexts and sometimes for multiple contexts. They addressed the contexts of behaviour change (11 approaches), public health and health promotion (9), digital health (6), complex interventions (5), quality or service improvement (3), clinical research (2), social policy or innovation (2) and others (Table 2).

### Key actions

The actions from all the included approaches were synthesised to identify a total of 18 actions. These actions are displayed in seven domains of intervention development. Although some authors describe intervention development as a broadly sequential process, in that some actions are usually undertaken prior to others [24], authors also emphasise that intervention development is a cyclical or iterative rather than linear process (for example, action four may generate understanding that takes developers back to action two), or there may be repetition within a single action until the developers are ready to move on to the next action [15, 17, 25–31]. Some actions may be undertaken concurrently. Therefore, the actions are presented within domains, with some attention to broad sequencing of domains and actions, to facilitate understanding of the process of intervention development. The seven domains are presented in three tables (Tables 3, 4 and 5) and described below.

**Table 3:** Synthesis of actions in conception and planning (based on all approaches in taxonomy)

Domain	Action	Methods
1. Conception	1. Identify that there is a problem in need of a new intervention [28, 29, 33, 34]	Authors of stepped or phased approaches to intervention development start by describing how a problem has been identified. The existence of a problem may be identified from published evidence synthesis, clinical practice, political strategy or needs assessment [28]. Alternatively, researchers or practitioners may have worked in a field for many years and identified the need for a new intervention [29]. In a clinical setting, the clinical significance of the problem, and the ability to make a clinically significant difference, is identified as the driver for selection of problems in need of a new intervention [34].
2. Planning	2. Establish a group or set of groups to guide the development process, thinking about engagement of relevant stakeholders such as the public, patients, practitioners and policy makers [27, 31, 34, 39–41, 53, 54]	Authors of a range of categories of intervention development explicitly consider the number, membership and role of groups that need to be established and run throughout the whole development process. Some authors recommend that a group is established that has ‘editorial rights’ (that is, makes final decisions about the intervention) and other groups are established that may deliver any technical expertise needed or offer advice and expertise for decision-making [31]. The ‘editorial rights’ group—sometimes called ‘the development team’—includes the developers and, in some approaches, includes members of the target population at which the intervention is aimed and practitioners likely to deliver it. Authors of user-centred approaches recommend including a variety of disciplines and expertise in this development team to generate innovation [33, 43]. Authors of stepped or phased approaches also recommend diverse membership to facilitate the development process, e.g. include people with computer science skills when designing digital interventions [34, 53]. In partnership approaches, the development team includes a diverse range of stakeholders, particularly members of the target population, who are equal partners with other team members, that is, have editorial rights [39, 40]. Those leading the intervention development will make efforts to encourage engagement of members of the target population, especially of hard-to-reach groups, develop inclusive communication processes for the group, and consider the assets (knowledge, experience, skills and abilities, influence and connections) available within the group [40]. This focus on bringing a variety of stakeholders together, and collaborative working with the target population and those who will deliver the intervention, is not unique to partnership approaches. Authors of some theory and evidence based approaches value this, working with a ‘planning group’ throughout the process, and seeking consensus after open discussion of diverse views [27]. Membership of these groups may change over time as the intervention, its target population, and who will deliver it become clear [24]. However, a unique aspect of partnership approaches is that members of the target population have decision-making rights throughout the development process.
	3. Understand the problems or issues to be addressed	Different authors address this action in different ways (see below). For partnership and target population-based approaches the focus is on in-depth understanding of the target population and the context in which the intervention will be delivered. For theory and evidence-based approaches this understanding is gained from theory and published research. Some approaches include both of these strategies but may place different weights on them. There are five sub-actions (i)-(v).
	(i) Understand the experiences, perspectives and psycho-social context of the potential target population The target population may be clients, patients, staff or a combination of these. This can involve identifying the priorities and needs of the potential target population, what matters most to people rather than what is the matter with them, why people behave as they do and understanding the lived experience of the potential target population [17, 25, 27, 30, 33, 34, 39–42, 44, 45, 53, 54, 56]	Some authors highlight this as the first action in the process and one that shapes the whole process [30, 33]. It is central to partnership and target population centred approaches where understanding the lived experiences and needs of the target population is the basis of the intervention. Secondary and primary qualitative research is recommended: synthesis of qualitative research; iterative qualitative research using diverse samples and open questions to explore people’s experiences and needs; use of patients’ narratives or archives of patient experiences and observation; consultation with stakeholders; and use of patient and public involvement [17, 41, 42]. Use of observation or ‘shadowing’ patients and families is recommended as well as obtaining the views of the target population because people may not be able to articulate the problem fully [33, 40]. Theory and evidence-based approaches, and stepped or phased approaches, also make use of qualitative research with the target population, including observation [27, 34].
	(ii) Assess the causes of the problems This will include the determinants of these causes, influences on the problems, the size of problems and who will benefit most and least from any intervention [24, 27–29, 49]	Authors of a range of approaches recommend the use of the evidence base through literature or systematic reviews [24, 29, 34]. Alternatives are drawing a logic model of the problem or model of causal pathways [27, 28] and creative approaches, such as group discussions, as a way of developing questions for research evidence reviews [27].
	(iii) Describe and understand the wider context of the target population and the context in which the intervention will be implemented Consider context at different levels: macro, meso, micro. Consider this context throughout the process [7, 17, 26, 27, 33, 44–46, 53, 56]	This sub-action can be undertaken as part of the earlier sub-actions (i) and (ii) but some approaches emphasise the importance of understanding context and so it is described as a separate action here. Bartholomew specifies the contexts of population, setting and community [27]. Again, the use of qualitative research, particularly observation, is recommended. The observation may be of service delivery where the intervention will occur [41, 42] or of the target population in their real life context [33, 43–45]. Conducting an asset assessment, that is, determining the strengths of the community in which an intervention will take place is useful for a health promotion intervention [27]. Some theories can help to understand important aspects of context for implementation of the intervention in the real world [46].
	(iv) Identify evidence of effectiveness of interventions for these problems, or for similar interventions once decisions have been made about the intervention type, so do not reinvent the wheel. Understand why previous interventions failed so can learn from this [7, 17, 31]	A range of approaches recommend systematic reviews of quantitative evidence of effectiveness of interventions to identify what has worked, and qualitative evidence to understand why interventions have worked or not [7, 17, 31].
	(v) Understand wider stakeholders’ perspectives of the problems and issues [24, 28, 29, 39, 40, 59]	Authors of partnership and stepped/phased approaches recommend working with wider stakeholders such as policy makers, community leaders or service providers to clarify and understand the problems. This can involve using research methods to obtain their views, meetings to facilitate communication, or equal partnership with stakeholders using activities to encourage active engagement in the context of partnership approaches. Wider stakeholders may already be fully engaged within partnership approaches or because they are members of groups established in Action 2.
	4. Make a decision about the specific problem or problems that an intervention will address, and the aims or goals for the intervention. This may involve defining the behaviours to target [27, 56]	If a list of problems has been identified then decisions will need to be made about which to prioritise and focus on [27, 56].
	5. Identify possible ways of making changes to address the problems. This involves identifying what needs to change, how to bring about this change and what might need to change at individual, interpersonal, organisational, community or societal levels [7, 17, 26, 27, 29, 30, 34, 48, 55]	This action is addressed differently depending on the category of approach, and aim and context, of the intervention. Interventions aiming to address behaviour change in public health specify this action in detail, recommending the creation of a ‘logic model for change’ showing mechanisms of change and causal relationships between theory and evidence-based change methods [27, 28]. The emphasis is on drawing on existing theory or theories, and the research evidence base, to link determinants of a problem and the objectives of the intervention [27]. Identifying a variety of theories rather than a single theory, including theories relevant to later parts of the development process, e.g. implementation theory, is recommended [27] at this action. Other approaches offer less detail about how to do this but suggest drawing a ‘conceptual map’ [26] or point out that it should be influenced by the earlier qualitative research with stakeholders, including the target population and those who will deliver intervention [30]. Qualitative research can be used to ask why people would make any proposed changes, how change should occur and barrier and facilitators to change.
	6. Specify who will change, how and when. Selections may depend on consideration of the likely impact of the change, how easy it is to change, how influential it is for the problem being addressed, and how easy it is to measure [26, 27, 47]	Authors of theory and evidence-based approaches detail this action, recommending using the combination of a theory or theoretical framework with data from multiple sources such as interviews, focus groups, questionnaires, direct observation, review of relevant documents, literature and involvement of stakeholders such as staff or patients [26]. There may be a long list of issues to change and these will need to be prioritised at this action [26].
	7. Consider real-world issues about cost and delivery of any intervention at this early stage to reduce the risk of implementation failure at a later stage [7, 24, 27, 29, 33, 44–46, 48]	Understanding the context (see Action 3.iii above) can help here. Authors recommend considering wider issues such as the cost of an intervention or the stigma attached to using it [33] or how it fits with current expectations of a professional group that would deliver it [24]. This is a key action for implementation-based approaches. The authors of [48] recommend consideration of the barriers to reaching the target population, how the intervention will function for different sub-groups, what percentage of organisations would be willing to adopt the intervention when tested and ability to overcome any barriers [48]. This thinking and planning occurs early in the process and can involve formative research with wider stakeholders. Authors of a range of other approaches propose that implementation is considered at this early stage [24, 29], with the use of theory to facilitate understanding of this [46] and the need to keep implementation issues in mind throughout later processes [27]. Issues other than effectiveness and cost-effectiveness that are related to implementation can be considered: affordability, practicability, acceptability, safety and equity [26]. Authors of partnership approaches recommend bringing staff and patients together to increase engagement and improve implementation [42]. Authors of stepped or phased approaches recommend that developers have to understand the real world of practice so they can develop not only effective interventions but interventions that practitioners adopt [24].
	8. Consider whether it is worthwhile continuing with the process of developing an intervention [7, 48]	The cost of delivering an intervention may outweigh the benefits it can potentially achieve. This issue is addressed in economic modelling undertaken alongside RCTs but can also be considered at the planning step by modelling processes and outcomes to determine if it is worth developing an intervention [7]. Alternatively, if solutions to barriers to future implementation in the real world (see Action 7) cannot be found then it might not be worth developing an intervention [48].

**Table 4:** Actions within designing and creating (based on all approaches in taxonomy)

Domain	Action	Methods
3. Designing	9. Generate ideas about solutions, and components and features of an intervention [7, 17, 25–27, 30, 32, 33, 39, 40, 42]	Ways of generating ideas for the intervention differ based on the category of approach to intervention development: Work with stakeholders creatively Partnership and target population-centred approaches recommend bringing together a number of groups (e.g. patients, service providers and product designers) to generate diverse ideas for solutions from different perspectives. This is the central tenet of a co-design approach where patients are equal partners in the whole process rather than simply having their views sought [39–41]. Authors of partnership approaches propose that listening to all voices is important, that processes to ensure that this is undertaken in a meaningful way may be needed [40] and that active engagement of diverse groups of stakeholders is ongoing throughout the whole process [25, 42, 44, 45]. Encouraging all members of the development team to interact directly with members the target population can guide the development of solutions that are more relevant and acceptable to the target population [30]. Methods to engage stakeholders may involve the use of games/exercises/tasks to promote creativity [25, 30, 33, 40] and the iterative use of prototypes (see step 4). Target population involvement in intervention development at this design domain is essential for authors of a range of approaches [7, 17, 25, 27, 30, 31, 42] with a proposal to make this involvement short and creative for busy people [25]. Starting with divergent thinking and moving to convergent thinking is proposed as a way of maximising the potential to identify the most powerful solutions [30].
		Use theory Theory and evidence-based approaches to intervention development recommend mapping behavioural determinants to behaviour change techniques. This is a key focus of the Behaviour Change Wheel, where lists of behaviour change techniques are given so that developers can identify intervention functions such as education or persuasion that can address the selected behaviours [26]. This is also a core action in Intervention Mapping with the construction of matrices to facilitate this action [27]. Matrices have been constructed by a group of experts for a specific behaviour change for a specific age group so that developers who are not experts in psychological theory can undertake this action [32]. Authors of some theory-based approaches advocate creative thinking as well as use of theory at this action [27]. A variety of theories rather than a single theory may be considered because one theory cannot explain everything of relevance [27]
	10. Re-visit decisions about where to intervene This can involve consideration of the different levels at which to intervene, and the wider system in which the intervention will operate [7, 17, 24, 26–29, 34, 54–56]	Consideration of where to intervene starts earlier at Action 4 but at this point final decisions need to be made. The authors of some approaches propose that this will require several team meetings but they are not always clear about who should be involved in these meetings. The ‘planning group’ [27] or ‘editorial group’ [31] may do this. Decisions are made about: • the scope of the intervention • the target population (this may be narrower or broader than at the Conception and Planning steps) • levels at which the intervention is aimed: individual, community or population • key features of the intervention (which may be components of other interventions) • the components that will address the change required • the amount of exposure needed to obtain effect
	11. Make decisions about the content, format and delivery of the intervention [24, 26–28, 30, 31, 47, 48, 54, 55]	Ideas generated in Action 9 are prioritised for inclusion in the intervention. Decision-making can be guided by the involvement of stakeholders, and theory and evidence including theories on what motivates people to engage in processes as well as produce outcomes, and use of taxonomies of modes of delivering interventions and evidence of effectiveness of these modes. Spencer [40] recommends using small ‘action groups’ of stakeholders who can use their relevant expertise to build the solution to the problem identified. Feasibility, budget and time constraints can inform choices [27]. Authors of only one approach recommend a formal consensus exercise for decisions in the specific case where published research evidence is summarised within the intervention [31]. Issues identified for consideration here, based on a range of different approaches, include: • what will be delivered (content) • who will deliver it • where it will be delivered • how it will be delivered (format) • how many times it will be delivered • the point in any treatment or illness trajectory it will be delivered • the order in which different parts of the intervention will be delivered • the time period over which it will be delivered • interactions between components • how users will be recruited • the resources available for delivery • how implementers will be trained • the potential for harm • the meaning of fidelity
	12. Design an implementation plan, thinking about who will adopt the intervention and maintain it [27, 48]	Consideration is given to implementation at the Planning domain (Action 7 earlier) but this action relates to establishing a formal implementation plan once the content, format and delivery of the intervention is known. Some authors recommend that this plan is based on the formative research undertaken earlier to understand barriers to implementation [48]. Others recommend basing this plan on a combination of theory and evidence from implementation science, and participation of stakeholders, to promote the use of the intervention in the real world [27]
4. Creating	13. Make prototypes or mock-ups of the intervention, where relevant [17, 25, 27, 30, 31, 33, 39, 40, 53, 54, 56, 59]	This action starts in the Design domain, and indeed is seen as an essential action in the Design domain by authors of some approaches. It is identified as a separate domain here because it is identified as such a key part of the process of intervention development by some authors. Testing prototypes can help developers to make decisions about the content, format and delivery of the intervention. It also continues into the Refining domain where refinements are made to prototypes as feasibility and acceptability is assessed. Authors of approaches to digital interventions recommend creating an early prototype of any physical intervention to get feedback from the target population using think aloud, usability testing, interviews or focus groups [17, 30, 57]. The prototype can be rough, e.g. paper copies of what a digital application could look like, and can be changed rapidly after feedback from stakeholders. These prototypes can generate further ideas for different prototypes as well as refine a current prototype. In some user-centred and digital approaches, authors recommend producing multiple rough and cheap prototypes at first and then reducing these down to a single prototype after stakeholder feedback [30, 33, 59]. They do this rather than focus on a single prototype too soon and call them ‘build to think’ prototypes [59]. If commercial designers are involved in creating prototypes, then differences in language and values between academia and commercial designers will need to be discussed, a contract established and decisions defined [53]

**Table 5:** Actions in refining, documenting and planning for future evaluation (based on all approaches in taxonomy)

Domain	Action	Methods
5. Refining	14. Test on small samples for feasibility and acceptability and make changes to the intervention if possible [17, 24, 27, 28, 30, 31, 34, 53, 54, 57]	Authors of a range of approaches recommend iterative testing and formative evaluation for this action. Some recommend qualitative research with those receiving and delivering the intervention. For example, think aloud interviews with the target population as they use the intervention, videos of people using the intervention [17, 30, 31, 57] or asking users to keep a diary of issues arising when using the intervention to prompt their memory during interviews. For some approaches the prototype discussed in the Creating domain is used to obtain specific views rather than general views on the intervention [31, 44, 45] and the use of observation moves beyond people’s views. The process can start small, for example with only the development team commenting on the first prototype and then widening the sample to members of the target population [31]. Quantitative as well as qualitative research is recommended, for example pre-test post-test comparison looking for changes in some intermediate outcomes for a small number of the target population using the intervention [34]. The potential for unintended consequences can also be considered [48].
	15. Test on a more diverse population, moving away from the single setting where early development of the intervention took place and seeking a more diverse sample. This can involve asking questions such as ‘is it working as intended?’, ‘is it achieving short term goals?’, ‘is it having serious adverse effects’? [17, 24, 25, 28, 31, 34, 54, 57]	The iterative approach used in Action 14 continues here by making changes to the intervention and continuing to use mixed methods to check if changes are working as planned on more diverse samples. Authors of a range of approaches recommend using pre-test post-test design, n-of-1 trials and observation or video to consider acceptability and early feasibility. They also recommend using real members of the target population in a real-life environment to identify interactions and relationships between different service providers and patients to iteratively modify the intervention. Groups of wider stakeholders can review the intervention as it iterates [24, 54].
	16. Optimise the intervention for efficiency prior to full RCT [34, 49–52]	Some approaches consider Actions 14 and 15 to be part of the process of optimisation of the intervention through the use of mixed methods. Case series can be used to consider issues such as dose, patterns of use over time, and safety [34]. Efficiency-based approaches and some stepped/phase approaches take a more quantitative approach: fractional factorial designs can be used to identify active components, interactions between components of an intervention, the doses that lead to best outcomes and tailoring to sub-groups [34, 50]. A review of optimisation of interventions has been published [4].
6. Documenting	17. Document the intervention, describing the intervention so others can use it and offer instructions on how to train practitioners delivering the intervention and on how to implement the intervention [7, 17, 24, 26, 29, 34, 48, 57]	This document is sometimes called a manual. The manual is written by the developers. Authors of some approaches recommend that it undergoes external review by stakeholders, including the target population and those delivering the intervention, to make sure it is feasible for use in the real world [24, 26, 29].
7. Planning for future evaluation	18. Develop the objectives of the outcome and process evaluations. This includes determining how outcomes and mediators of change can be measured, developing measures, specifying evaluation design, planning recruitment and considering feasibility of a full RCT [24, 27, 29, 31, 40, 47, 48]	Authors of some approaches recommend planning for a randomised study or experimental design with controls for measuring effectiveness [29], whereas others recognise that there may not always be the resources to evaluate with a RCT so the intervention may be implemented in practice and monitored [31]. Authors of some approaches recommend involving stakeholders such as funders, implementers and the target population in this action [27], particularly getting agreement with key stakeholders about how to define and measure success [48]. Partnership approaches recommend that this is undertaken with stakeholders [40].

Authors of some approaches state that intervention development is a cumulative or progressive process, arguing that it is necessary to spend time on the early actions and get this right because later ones depend on these [26].

#### Conception and planning

The first two domains of intervention development are Conception and Planning (Table 3). Although the Conception domain has only one action, it emphasises the importance of being transparent about where the idea for an intervention has originated. The Planning domain has seven actions, from deciding who will be involved in the development process and how, through to considering whether it is worthwhile designing an intervention (Table 3). Authors of some approaches identify the need to spend resources on the Planning domain to get the intervention right (e.g. [32]).

#### Designing and creating

The next two domains of intervention development are Designing an intervention and then Creating it (Table 4). The Creating domain can be an integral action within the Designing domain in some approaches. Two ways of addressing both of these domains have been proposed: one prioritises working with stakeholders, particularly the target population, and the other focuses on theory. Constructing a rough prototype early in these domains is key to some approaches. These domains require creativity [24, 33, 34], with use of a multidisciplinary team proposed as a way to maximise idea generation and innovation [30, 34].

#### Refining, documenting and planning for future evaluation

The final three domains are Refining, Documenting and Planning for Future Evaluation (see Table 5). The Refining domain starts by testing early versions of the intervention on a small sample, and asking whether it merits more rigorous and costly testing [34] before moving to testing on a diverse sample to improve external validity [17, 27]. Some approaches consider this domain to be part of the process of optimising the intervention and propose the use of mixed methods research as necessary [17, 27]. Other approaches propose quantitative experimental designs for optimising the intervention. A number of approaches recommend iterative processes, as new insights emerge and changes are made to the intervention.

The Documenting domain involves writing a set of instructions so others can use the intervention. The compilation of this document or manual is likely to start early in the process and be finalised towards the end of the development process. The document or manual, like the intervention, is likely to undergo multiple iterations during the development process and be further refined after any formal pilot or evaluation. The description of the intervention in any document can follow guidelines for reporting interventions: why, what, who provides, how, where, when and how much, and tailoring [35].

Some approaches recommend planning for an eventual evaluation from the beginning of the development process [24, 27].

### Methods and activities used in the development process

Authors of different approaches propose using different research methods and activities to deliver each action (Tables 3, 4 and 5). Methods and activities include using non-participant observation to understand the problem and the context in which the intervention will operate, playing games to generate ideas for the content of the intervention and using ‘think aloud’ methods to understand the usability, acceptability or feasibility of early versions of the intervention.

### Strengths and limitations of approaches

The strengths of each approach, reported largely by its authors, included those related to processes (that they are systematic, practical, clear or detailed) and outcomes (that they have been used a number of times, or there is evidence that they have produced effective interventions). Authors did not formally compare their approach with others. Limitations, again reported largely by authors, included that some approaches lacked detail, were time consuming to undertake, required expertise or did not address how to deal with conflicting opinions from diverse stakeholders. These strengths and limitations are further detailed below.

#### Comprehensiveness

Some of the approaches were more comprehensive than others in addressing a higher proportion of the 18 actions (Table 6). More comprehensive approaches included Intervention Mapping and the Behaviour Change Wheel. Some offered more detail about how to undertake specific actions because they were published as books or included a lot of examples. These included Intervention Mapping, the Behaviour Change Wheel and the Person-Based Approach. There appeared to be a tension between offering detailed description and being too prescriptive.

**Table 6:**
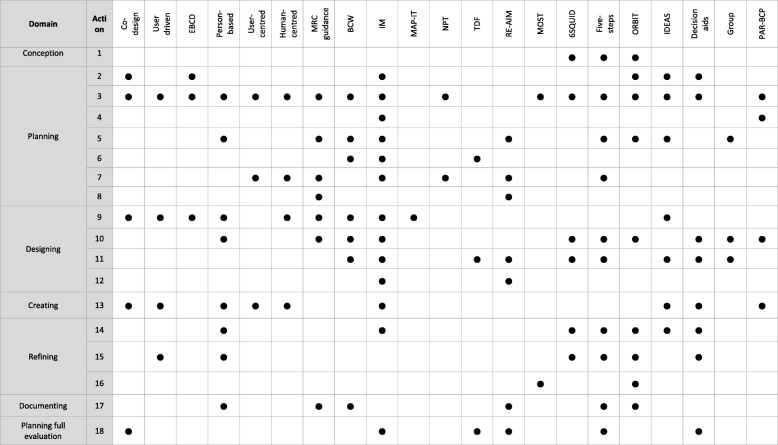
Actions undertaken in intervention development within each approach (black dot in a cell indicates that an action labelled 1–18 described in Tables 3, 4 and 5 is recommended by an approach)

#### Utility or success

Some authors reported how their approach had been used to develop interventions that were found to be effective. Such approaches included Experience Based Co-design, Intervention Mapping, the Behaviour Change Wheel and Person Based Approach.

#### Resource and expertise

Some authors explicitly addressed concerns about the resource needed to develop an intervention. Some had undertaken a part of one approach that required expertise and constructed a new approach to reduce the cost and knowledge required (MAP-IT). Some proposed that their approach could be completed with limited resources by reducing the length of time or resource spent on each action (Intervention Mapping). One proposed that the time and resources allocated should be influenced by the importance of the problem being addressed (Behaviour Change Wheel).

#### Relationship between approaches

Most approaches were proposed as independent ways of developing interventions. Some were proposed as complementary to existing approaches (Person-Based Approach), as explicit combinations of existing approaches (PAR-BCP) or as ways of facilitating existing approaches (MAP-IT). Some included the same actions but were in different categories of the taxonomy because of the weight placed on those actions in the stated rationale.

## Discussion

### Summary of findings

This is the most comprehensive review and synthesis of diverse approaches to intervention development undertaken to date. Eight categories of approach were identified and descriptive summaries of approaches within each category offered. There is considerable overlap between categories in terms of the actions required, although there are differences in the methods that authors of different approaches use to address each action. Some approaches are more comprehensive than others in terms of addressing a wide range of actions, and some offer more detailed accounts than others to help researchers develop their own interventions. Intervention development is a rapidly developing field and recent additions have been proposed as complementary to existing approaches [17] or as enhancements [10].

### How to use the taxonomy and synthesis of actions

This overview presents a broad range of approaches and actions common to intervention development, to help developers to select the approach or actions to suit their context, values and needs. Intervention developers should consider the following questions when deciding how to develop an intervention:

1. What is the intention of the intervention? e.g. changing behaviour
2. What is the context of the intervention? e.g. public health
3. What values inform the intervention development? e.g. working in partnership with the target population
4. What skills and experience does the team bring?
5. Which approaches have resulted in interventions shown to be effective?
6. What resources are available for the intervention development?

Some of these issues can be considered by reading Table 2 where the different approaches are summarised along with their contexts, strengths and limitations, and Table 6 which shows which approaches address the different actions reported in this overview.

The synthesis of actions from all the approaches shows the range of issues to consider. It may not be possible to undertake all these actions, and indeed not all of them may be necessary. By laying out the range of actions in this overview, researchers can consider the possibilities available to them and which approaches address the actions they value for their specific context (see Table 6).

### Placing the findings in the context of other research

The approaches identified in other more narrowly focused reviews of intervention development are included in this overview, for example MRC Guidance, Intervention Mapping and multiphase optimization strategy (MOST) [3, 4]. One of the earlier reviews identified four tasks of intervention development which are included in the actions identified in this overview: identifying barriers, selecting intervention components, using theory and engaging end-users [3]. A review of the success of different types of interventions in diabetes in childhood assessed the scientific rigour of the development processes of each type of intervention [36]. The checklist for scientific rigour was based on the content of one approach to intervention development, and reporting guidelines. Items in this checklist appear in the actions in this overview, e.g. existence of a manual to guide procedures, and explicit statement of theoretical basis of the intervention. A recent paper was published on enriching the development phase of the MRC Framework by including steps from seven other approaches [10]. Again, the steps identified are included in this overview: problem identification, systematic identification of evidence, identification or development of theory, determination of needs, examination of current practice or context and modelling process and expected outcomes.

### Strengths and limitations

This is the first time such a broad and detailed review of approaches to intervention development has been undertaken. The overview is timely given the current interest in undertaking good intervention development. There were five limitations. First, there was a subjective aspect to the selection of approaches that is common to all qualitatively oriented reviews. Team discussion was used to address subjectivity and a description has been given of how decisions were made by authors of this overview. It is likely that the majority—but not all—of the included approaches would appear in a similar overview undertaken by a different team. Second, as planned, this is not an exhaustive list of approaches and readers will be able to name other approaches. Third, the taxonomy was based on the rationale provided by authors of approaches. This rationale was not always explicit, and was dependent on the report given in the websites, books or articles included; authors of approaches may not necessarily agree with the interpretation of their rationale. Fourth, this is a rapidly developing field and new approaches were published after the end of formal data extraction and were not included [10, 37]. Finally, a comparison of the success of each approach has not been undertaken (for example, the number of times effective interventions have been developed using each approach), although authors’ own reports of success have been included. Further research could synthesise evidence of effectiveness of different categories of approach, specific approaches or actions within approaches.

### Research gaps

Another important part of the picture is to understand how interventions are actually developed in practice. A review of primary research reporting this activity is being undertaken as part of the wider INDEX study. The intention is to compare the approaches reported here with practice. It is already apparent that some approaches are used in primary studies but documentation describing the approach in the context of intervention development could not be found for inclusion in this overview, in particular community-based participatory research. The use of patient and public involvement within intervention development is also absent from the approaches described here. This may be more prominent in the review of primary research studies in the INDEX study noted above.

## Conclusions

This overview of approaches to intervention development can help researchers to understand the variety of approaches available and the range of possible actions involved in intervention development, before undertaking any feasibility or piloting phase. Findings from this overview will contribute to future guidance on intervention development.

## Additional file

Additional file 1: Search strategy. (DOCX 21 kb)

## Abbreviations

6SQUID: Six essential Steps for Quality Intervention Development
BCW: Behaviour Change Wheel
EBCD: Experience-based co-design
IDEAS: Integrate, Design, Assess and Share
IM: Intervention mapping
INDEX: IdentifyiNg and assessing different approaches to DEveloping compleX interventions
MAP-IT: Matrix Assisting Practitioner’s Intervention Planning Tool
MOST: Multiphase optimization strategy
MRC: Medical Research Council
NPT: Normalisation process theory
ORBIT: Obesity-Related Behavioural Intervention Trials
PAR-BCP: Participatory Action Research based on theories of Behaviour Change and Persuasive Technology
RE-AIM: Reach, Effectiveness, Adoption, Implementation, Maintenance
TDF: Theoretical domains framework
UK: United Kingdom
