# Supplementary material for: Taxonomy of approaches to developing interventions to improve health: a systematic methods overview
Source: Pilot Feasibility Stud. 2019 Mar 12;5:41. doi: 10.1186/s40814-019-0425-6 (PMC6419435; doi:10.1186/s40814-019-0425-6)
Supplement: Supplementary file 1 — Search strategy. (DOCX 21 kb) [file 40814_2019_425_MOESM1_ESM.docx]

**Additional file 1 Search strategy**

**Primary search**

MEDLINE Epub Ahead of Print, In-Process & Other Non-Indexed Citations, Ovid MEDLINE(R) Daily and Ovid MEDLINE(R)

3^rd^ January 2017

| **#** | **Searches** |
| --- | --- |
| 1 | intervention development.ti,ab. |
| 2 | limit 1 to english language |
| 3 | limit 2 to yr="2015 - 2016" |

CINAHL

3^rd^ January 2017

| **#** | **Searches** |
| --- | --- |
| 1 | TI intervention development OR AB intervention development Limiters - Published Date: 20150101-20161231; English Language |

PsycINFO

3^rd^ January 2017

| **#** | **Searches** |
| --- | --- |
| 1 | intervention development.ti,ab. |
| 2 | limit 1 to english language |
| 3 | limit 2 to yr="2015 - 2016" |

ASSIA

3^rd^ January 2017

| **#** | **Searches** |
| --- | --- |
| S1 | ti("intervention development") OR ab("intervention development") Additional limits - Date: 2015-2016; Language: English |

ERIC

3^rd^ January 2017

| **#** | **Searches** |
| --- | --- |
| S1 | ti("intervention development") OR ab("intervention development") Additional limits - Date: 2015-2016; Language: English |

**Wider search to check breadth of primary search**

MEDLINE Epub Ahead of Print, In-Process & Other Non-Indexed Citations, Ovid MEDLINE(R) Daily and Ovid MEDLINE(R)

3^rd^ January 2017

| **#** | **Searches** |
| --- | --- |
| 1 | ((complex or behav?oural) adj3 intervention*).ti,ab. |
| 2 | develop*.ti,ab. |
| 3 | design*.ti,ab. |
| 4 | clinical trial, phase i.pt. |
| 5 | (phase adj ('1' or I or first or one)).ti,ab. |
| 6 | refine*.ti,ab. |
| 7 | translat*.ti,ab. |
| 8 | exploratory.ti,ab. |
| 9 | or/2-8 |
| 10 | 1 and 9 |
| 11 | limit 10 to yr="2015 - 2016" |
| 12 | limit 11 to english language |

CINAHL

3^rd^ January 2017

| **#** | **Searches** |
| --- | --- |
| S1 | TI ( ((complex or behav?oural) N3 intervention*). ) OR AB ( ((complex or behav?oural) N3 intervention*) ) |
| S2 | TI ( (develop* or design* or refine* or translat* or exploratory) ) OR AB ( (develop* or design* or refine* or translat* or exploratory) ) |
| S3 | TI ( (phase N1 ('1' or I or first or one)) ) OR AB ( (phase N1 ('1' or I or first or one)) ) |
| S4 | S2 OR S3 |
| S5 | S1 AND S4 |
| S6 | S1 AND S4 Limiters - Published Date: 20150101-20161231; English Language |

PsycINFO

3^rd^ January 2017

| **#** | **Searches** |
| --- | --- |
| 1 | ((complex or behav?oural) adj3 intervention*).ti,ab. |
| 2 | develop*.ti,ab. |
| 3 | design*.ti,ab. |
| 4 | (phase adj ('1' or I or first or one)).ti,ab. |
| 5 | refine*.ti,ab. |
| 6 | translat*.ti,ab. |
| 7 | exploratory.ti,ab. |
| 8 | or/2-7 |
| 9 | 1 and 8 |
| 10 | limit 9 to yr="2015 -2016" |
| 11 | limit 10 to english language |

ASSIA

3^rd^ January 2017

| **#** | **Searches** |
| --- | --- |
| S1 | (ti(((complex OR behav?oural) NEAR/3 intervention*)) OR ab(((complex OR behav?oural) NEAR/3 intervention*))) AND ((ti(develop* OR design* OR refine* OR translat* OR exploratory) OR ab(develop* OR design* OR refine* OR translat* OR exploratory)) OR (ti((phase N ('1' OR I OR first OR one))) OR ab((phase N ('1' OR I OR first OR one))))) AND (la.exact("ENG") AND pd(20150101-20161231)) |

ERIC

3^rd^ January 2017

| **#** | **Searches** |
| --- | --- |
| S1 | (ti(((complex OR behav?oural) NEAR/3 intervention*)) OR ab(((complex OR behav?oural) NEAR/3 intervention*))) AND ((ti(develop* OR design* OR refine* OR translat* OR exploratory) OR ab(develop* OR design* OR refine* OR translat* OR exploratory)) OR (ti((phase N ('1' OR I OR first OR one))) OR ab((phase N ('1' OR I OR first OR one))))) AND (la.exact("ENG") AND pd(20150101-20161231)) |
